# Supplementary material for: An Explainable Geometric-Weighted Graph Attention Network for Identifying Functional Networks Associated with Gait Impairment
Source: ArXiv. 2023 Jul 24:arXiv:2307.13108v1. Preprint. [Version 1] (PMC10402187)
Supplement: 1 [file NIHPP2307.13108V1-supplement-1.pdf]

Supplementary Material for "An Explainable Geometric-Weighted Graph Attention Network for Identifying Functional Networks Associated with Gait Impairment"

Riemmanian Metrics

The Riemannian metric is a geometric structure that assigns a positive-definite inner product to each tangent space of a smooth manifold. This allows us to define notions of length, angle, and curvature on the manifold. Let $\mathcal{M}$ be a smooth manifold, the Riemannian metric on $\mathcal{M}$ is a smoothly varying family of inner products g_p on the tangent spaces $\mathbf{T}_p\mathcal{M}$, for each point $p \in \mathcal{M}$.
Affine Invariant Riemannian Metric (AIRM): AIRM[1] is an intrinsic Riemannian metric that characterizes the geometry of a symmetric positive definite (SPD) space by returning a number signifying the geodesic distance between two elements in tangent space. Given two SPD matrices $\mathbf{P}$ and $\mathbf{Q}$, the AIRM distance is defined as:

$$d_{\mathrm{AIRM}}(\mathbf{P}, \mathbf{Q}) = \sqrt{\sum_{i=1}^{n} \log^2 \lambda_i}$$

where λ_i are the eigenvalues of $\mathbf{P}^{-1}\mathbf{Q}$. AIRM is invariant to affine transformations, which makes it robust in many applications, and is strictly bound by innate geometric conditions.
Log-Euclidean Riemannian Metric (LERM): LERM[2] is an extrinsic, closed-form formula for the Riemannian mean and is used to measure the geometric difference between matrices in a Riemannian manifold. Given two SPD matrices $\mathbf{P}$ and $\mathbf{Q}$, the LERM distance is defined as:

$$d_{\mathrm{LERM}}(\mathbf{P}, \mathbf{Q} = \|log(\mathbf{P}) - log(\mathbf{Q})\|_F^2$$

where $|\cdot|_F^2$ denotes the Frobenius norm. While not affine-invariant, LERM is particularly advantageous because it embeds points in SPD matrices into a Euclidean space, making them amenable to standard Euclidean distance computations while preserving geometric information and thus is more computationally efficient than affine-invariant metrics like AIRM.
***symmetrized* Kullback-Leibler Divergence Metric (sKLDM)**: sKLDM[3] is an extension of the asymmetric KL divergence metric (KLDM), which quantifies the dissimilarity between probability distributions on a Riemannian manifold while taking into account the symmetrical nature of their comparison. It is also known as Jeffreys

[1] Pennec, X., Fillard, P. and Ayache, N.: A Riemannian framework for tensor computing. International Journal of computer vision, 66, pp.41-66. (2006)
[2] Arsigny, V., Fillard, P., Pennec, X., Ayache, N.: Geometric means in a novel vector space structure on symmetric positive-definite matrices. SIAM journal on matrix analysis and applications 29(1), 328–347 (2007)
[3] Kullback, S. and Leibler, R.A.: On information and sufficiency. The annals of mathematical statistics, 22(1), pp.79-86. (1951)

Method	Pre	Rec	$\mathbf{F_1}$	AUC
**xGW-GAT** (AIRM)	0.70	0.67	0.61	0.67
**xGW-GAT** (sKLDM)	0.55	0.45	0.49	0.51
**xGW-GAT** (LERM)*	**0.75**	**0.77**	**0.76**	**0.83**

Table 1. Ablation study for Riemannian metrics: *Note that the LERM results are the same as our best, reported results in the main paper.

divergence. Given two SPD matrices $\mathbf{P}$ and $\mathbf{Q}$, the KLDM geodesic distance is defined as:

$$d_{\text{KLDM}}(\mathbf{P}, \mathbf{Q}) = Tr(\mathbf{P}(log(\mathbf{P}) - log(\mathbf{Q}))$$

where $Tr(\cdot)$ denotes the trace operator. The symmetrized function is as follows:

$$d_{\text{sKLDM}}(\mathbf{P}, \mathbf{Q}) = \frac{1}{2}\left(d_{\text{KLDM}}(\mathbf{P}, M) + d_{\text{KLDM}}(\mathbf{Q}, M)\right)$$

where M is the average of the two distributions, i.e. $M = \frac{1}{2}(\mathbf{P} + \mathbf{Q})$.

Sample Selection Node Centrality Measures

For each training sample, we derive the following node features that encode node centrality measures, i.e., a node's importance or influence within the network, based on criteria such as the number, quality, and proximity of its connections.

- *Degree Centrality*: Degree centrality of a node j, denoted $C_D(j)$, is the count of its direct connections or edges, defined as:

  $$C_D(j) = \deg(j)$$

  where $\deg(j)$ is the degree of node d.
- *Eigenvector Centrality*: Eigenvector centrality of a node j, denoted $C_E(j)$, measures its influence based on the quality of its connections, defined as:

  $$C_E(j) = \frac{1}{\lambda} \sum_{i \in N(j)} A_{ij} C_E(i)$$

  where A_{ij} is the adjacency matrix, $N(j)$ is the set of neighbors of i, and λ is the largest eigenvalue.
- *Closeness Centrality*: Closeness centrality of a node v, denoted $C_C(j)$, measures the inverse average shortest path length to all other nodes, defined as:

  $$C_C(j) = \frac{1}{\sum_{u \neq v} d(j, i)}$$

  where $d(j, i)$ is the shortest path length from j to i.
